# Optimal cut-point definition in biomarkers: the case of censored failure time outcome

**DOI:** 10.1186/s12874-015-0009-y

**Published:** 2015-03-21

**Authors:** Matteo Rota, Laura Antolini, Maria Grazia Valsecchi

**Affiliations:** Department of Health Sciences, Centre of Biostatistics for Clinical Epidemiology, University of Milan-Bicocca, Monza, Italy

**Keywords:** Optimal cut-point, Censored failure time outcome, Youden index, Concordance probability, Point closest-to-(0,1) corner in the ROC plane

## Abstract

**Background:**

Cut-point finding is a crucial step for clinical decision making when dealing with diagnostic (or prognostic) biomarkers. The extension of ROC-based cut-point finding methods to the case of censored failure time outcome is of interest when we are in the presence of a biomarker, measured at baseline, used to identify whether there will be the development, or not, of some disease condition within a given time point τ of clinical interest.

**Methods:**

Three widely used cut-point finding methods, namely the Youden index, the concordance probability and the point closest to-(0,1) corner in the ROC plane, are extended to the case of censored failure time outcome resorting to non-parametric estimators of the sensitivity and specificity that account for censoring. The performance of these methods in finding the optimal cut-point is compared under Normal and Gamma distributions of the biomarker (in subjects developing or not the disease condition). Normality ensures that estimators point theoretically to the same cut-point. Two motivating examples are provided in the paper.

**Results:**

The point closest-to-(0,1) corner approach has the best performance from simulations in terms of mean square error and relative bias.

**Conclusions:**

We discuss the use of the Youden index or concordance probability associated to the cut-point identified through the closest-to-(0,1) corner approach to ease interpretability of the classification performance of the dichotomized biomarker. In addition, the achieved performance of the dichotomized biomarker classification associated to the estimated cut-point can be represented through a confidence interval of the point on the ROC curve.

**Electronic supplementary material:**

The online version of this article (doi:10.1186/s12874-015-0009-y) contains supplementary material, which is available to authorized users.

## Background

The use of a continuous biomarker X in clinical practice often requires the definition of a cut-point c above (or below) which subjects are classified, for instance, as diseased and disease-free. In the presence of a binary outcome, methods based on the receiver operating characteristic (ROC) curve are commonly and indistinctly used. These methods are based on objective functions of c:

i) the Youden function, defined as the difference between the probability of X > c in diseased subjects (sensitivity, SE) and the complement to one of the probability of X ≤ c in disease-free subjects (specificity, SP), i.e. SE + SP-1. The chosen c maximizing this function, or equivalently SE + SP, leads to a maximum value known as Youden index [1];
ii) the concordance probability function, equal to the product of SE and SP, where the chosen c maximizes this function [2,3];
iii) the distance between the point (1-SP, SE) and the optimal point (0,1) in the ROC plane [4], where the chosen c leads to the minimum distance, and the operating point is referred as point closest-to-(0,1) corner.

A recently published work compared these methods by simulation in the case of a binary outcome [5]. The authors showed that the point closest-to-(0,1) corner [4] and concordance probability [2,3] methods outperformed both the Youden index [1] and the minimum P-value approaches [6].

The extension of ROC-based cut-point finding methods to the case of censored failure time outcome is of interest when we are in the presence of a biomarker, measured at baseline in a cohort of disease-free subjects, and used to understand whether there will be the development, or not, of a disease condition within a given time point τ of clinical interest. However, this extension is not straightforward. In fact, both SE and SP cannot be estimated by simple proportions as in the case of a binary outcome, because it is not known whether censored subjects should be considered as diseased or disease-free up to τ. As a consequence, a suitable estimator for SE and SP need to be used to account for the presence of censoring.

We aimed to extend the Youden index, concordance probability and point closest-to-(0,1) corner cut-point estimation methods to the case of censored failure time outcome. The performance of the aforementioned methods in finding the optimal cut-point is compared under Normal homoscedastic and Gamma distributions of the biomarker in diseased and disease-free subjects [7]. Normality ensures that estimators point theoretically to the same cut-point, as previously shown and described [2,5].

For each method, the optimal cut-point is empirically estimated by maximization of objective functions [8] using the estimators for SE and SP derived in Antolini and Valsecchi [9]. To illustrate the methodology, two application examples are provided, one in an observational study of a molecular biomarker in acute lymphoblastic leukemia [10], and one concerning the definition of a prognostic score for patients with primary biliary cirrhosis enrolled in a randomized clinical trial [11].

## Methods

### Notations and basics

For a generic subject, let Z be the survival time, defined as the time elapsed between some initial time point, where the subject is disease-free, and the development in time of disease. Let τ be a time horizon of clinical interest. The definition of disease and disease-free conditions depends on whether Z ≤ τ or Z > τ. It is assumed that increasing values of the biomarker X are related to a possible increment of the risk of becoming diseased. Otherwise, without loss of generality, take the negative of X. For any cut-point c that defines a binary classification rule, a generic subject is said to be testing positive or negative depending on whether X > c or X ≤ c.

In this context, SE and SP at c are defined as the probability of testing positive given that the subject is diseased

1 $$ \mathrm{S}\mathrm{E}\left(\mathrm{c}\right)\kern0.5em =\kern0.5em \mathrm{P}\left(\mathrm{X}>\mathrm{c}{\left|\mathrm{Z}\right.}_{\mathrm{i}}\le \uptau \right) $$

and as the probability of testing negative given that the subject is disease-free

2 $$ \mathrm{S}\mathrm{P}\left(\mathrm{c}\right)\kern0.5em =\kern0.5em \mathrm{P}\left(\mathrm{X}\le \mathrm{c}\left|{\mathrm{Z}}_{\mathrm{i}}\right.>\tau \right). $$

**Figure 1:**
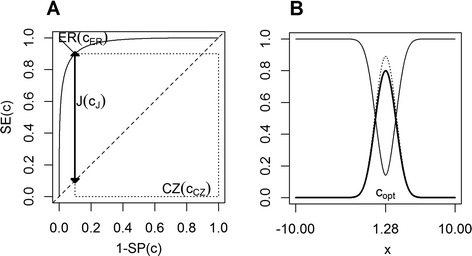
Cut-point finding methods background. Panel **A**. ROC-based objective functions. J(c_J_) is the thick line segment. CZ(c_CZ_) is the area of the dotted rectangle and ER(c_ER_) is the thin line segment. Panel **B**. Behaviour of the population objective functions J(c) (thick line), CZ(c) (dotted line) and ER(c) (thin line). CZ(c) is multiplied by 1.10. In the two panels, the biomarker X is generated according to N(2.56,1) or N(0,1), depending on whether Z ≤ τ or Z > τ, respectively, leading to J = 0.8, CZ = 0.81, ER = 0.14.

The ROC curve is defined as the plot of SE(c) across 1-SP(c), for varying c. It is represented in Figure 1, panel A.

The Youden function of c is the difference between SE(c) and 1-SP(c):

3 $$ \mathrm{J}\left(\mathrm{c}\right)\kern0.5em =\kern0.5em \mathrm{S}\mathrm{E}\left(\mathrm{c}\right)+\mathrm{S}\mathrm{P}\left(\mathrm{c}\right)-1. $$

J(c) takes values between 0, when SE(c) = 1-SP(c), and 1 when SE(c) = SP(c) = 1. The behavior of (3) is represented in Figure 1 panel B, thick line segment. The Youden index J [1] is defined as the maximum of the Youden function (3), or equivalently of SE(c) + SP(c). Graphically, J represents the maximum vertical distance between the ROC curve and the diagonal chance line representing a useless biomarker. It can be also interpreted as the maximum net gain of the true positive fraction (SE) with respect to the false positive fraction, i.e. 1-SP (Figure 1 panel A thick line segment). The c maximizing (3) is the optimal cut-point.

The concordance probability function [2,3] of c is the product of SE(c) and SP(c):

4 $$ \mathrm{C}\mathrm{Z}\left(\mathrm{c}\right)=\mathrm{S}\mathrm{E}\left(\mathrm{c}\right)\cdot \mathrm{S}\mathrm{P}\left(\mathrm{c}\right). $$

CZ(c) ranges between 0 if either SE(c) = 0 or SP(c) = 0, and 1 in the ideal case where SE(c) = SP(c) = 1. CZ(c) could be also expressed as the area of a rectangle on the ROC curve of width SP(c) and height SE(c) and interpreted as probability of being below or beyond c for any random pair of disease-free and diseased subjects (Figure 1 panel A, dotted line). The behaviour of (4) is represented in Figure 1 panel B, dotted line. The optimal cut-point according to this method is the c that maximizes (4).

The objective function defined as the distance between the couple (1-SP(c), SE(c)) and the optimal point (0,1) – representing maximum specificity (SP = 1) and maximum sensitivity (SE = 1) - in the ROC plane (Figure 1 panel A, thin line segment) is obtained by applying the Euclidean distance

5 $$ \mathrm{E}\mathrm{R}\left(\mathrm{c}\right)\kern0.5em =\kern0.5em \sqrt{{\left(1\kern0.5em -\kern0.5em \mathrm{S}\mathrm{E}\left(\mathrm{c}\right)\right)}^2\kern0.5em +\kern0.5em {\left(1\kern0.5em -\kern0.5em \mathrm{S}\mathrm{P}\left(\mathrm{c}\right)\right)}^2}. $$

The behaviour of (5) is represented in Figure 1 panel B, thin line segment. The optimal cut-point according to this method is the c that mimimizes (5).

The three objective functions (3), (4) and (5) lead theoretically to the same cut-point c_opt_ when considering homoscedastic Normal distributions of the biomarker in diseased and disease-free subjects (Figure 1). A formal proof is showed in Liu [2].

### Optimal cut-point estimation

Let T_i_ = min(Z_i_,C_i_) be the observed time, where Z_i_ is the time to event (development of disease) and C_i_ the right censoring time, δ_i_ the censoring indicator (δ_i_ = 1 if T_i_ = Z_i_ and δ_i_ = 0 if T_i_ = C_i_) and X_i_ the biomarker value for subject i. Independence completely at random between Z and C is assumed as in the classical framework of survival analysis. In our setting, the biomarker X is measured at baseline in order to identify ahead in time subjects that will develop disease or not within τ. The observed data in a sample of size N is {(X_i_, T_i_, δ_i_); i = 1, …, N}, and it can be subdivided in three subgroups:

i) *Disease-free*, if T_i_ > τ regardless of δ_i_;
ii) *Diseased*, if T_i_ ≤ τ and δ_i_ = 1;
iii) *Censored by τ* while *Disease-free*, if T_i_ ≤ τ and δ_i_ = 0;

with cardinality ${\mathrm{n}}_{\mathrm{z}>\tau }={\displaystyle {\sum}_{\mathrm{i}=1}^{\mathrm{N}}\mathrm{I}\left({\mathrm{T}}_{\mathrm{i}}>\tau \right)},\;{\mathrm{n}}_{\mathrm{z}\le \uptau}={\displaystyle {\sum}_{\mathrm{i}=1}^{\mathrm{N}}\mathrm{I}\left({\mathrm{T}}_{\mathrm{i}}\le \uptau \right)}\kern0.5em {\updelta}_{\mathrm{i}}$ and ${\mathrm{n}}_{\mathrm{C}}={\displaystyle {\sum}_{\mathrm{i}=1}^{\mathrm{N}}\mathrm{I}\left({\mathrm{T}}_{\mathrm{i}}\kern0.5em \le \kern0.5em \uptau \right)\left(1\kern0.5em -\kern0.5em {\updelta}_{\mathrm{i}}\right)}$, respectively.

For the n_c_ subjects belonging to iii), the disease status by τ is unknown as it is not possible to know whether they would experience or not the disease within τ if censoring would not have occurred. This leads for any c of X to a classification 3×2 matrix of the type:

where $$ {\mathrm{n}}_{\mathrm{X}\le \mathrm{c}}={\displaystyle {\sum}_{\mathrm{i}=1}^{\mathrm{N}}\mathrm{I}}\left({\mathrm{X}}_{\mathrm{i}}\le \mathrm{c}\right) $$ and $$ {\mathrm{n}}_{\mathrm{X}>c}={\displaystyle {\sum}_{\mathrm{i}\kern0.5em =1}^{\mathrm{N}}\mathrm{I}}\left({\mathrm{X}}_{\mathrm{i}}>c\right) $$.

In this setting, SE(c) (1) and SP(c) (2) are not directly estimable from (6) since it is not known how the n_c_ subjects would contribute to the classification 2x2 matrix contrasting the disease status for all N subjects to the biomarker classification.

Nonparametric estimators of SE(c) (1) and SP(c) (2) can be derived following two different approaches, the direct or the indirect one, that were recently discussed and shown to be equivalent by Antolini and Valsecchi [9]. Direct estimation is based on an inverse probability weighting scheme applied to the counts of the groups of disease-free and diseased subjects in the classification matrix (6) (lines 1 and 2), and originates from the consideration that subjects with observed status are selected from the censoring process and can be weighted to represent the subjects that are censored (line 3 of matrix (6)). Indirect estimation relies on writing SE and SP in terms of quantities that are estimable from the available data in the presence of censoring, and on plugging-in the estimates.

A further approach, equivalent to the aforementioned ones, consists in estimating the expected number of events in each of the four cells of the 2×2 classification matrix contrasting the disease status for all N subjects to the biomarker classification, as follows:

The two survival estimates ${\widehat{\mathrm{S}}}_{\mathrm{X}>c}\left(\uptau \right)\kern0.5em =\kern0.5em \widehat{\mathrm{P}}\left(\mathrm{Z}>\uptau \left|\mathrm{X}>\mathrm{c}\right.\right)$ and ${\widehat{\mathrm{S}}}_{\mathrm{X}\le \mathrm{c}}\left(\uptau \right)\kern0.5em =\kern0.5em \widehat{\mathrm{P}}\left(\mathrm{Z}>\uptau \left|\mathrm{X}\le \mathrm{c}\right.\right)$ are simply obtained by the Kaplan-Meier method in the two samples as classified according to the biomarker value X.

SE(c) (1) and SP(c) (2) can now be directly estimated from matrix (7) by simple proportions as

8 $$ \widehat{\mathrm{S}\mathrm{E}}\left(\mathrm{c}\right)\kern0.5em =\kern0.5em \frac{\left(1-{\widehat{\mathrm{S}}}_{\mathrm{X}>c}\left(\uptau \right)\right)\cdot {\mathrm{n}}_{\mathrm{X}>c}}{\left(1\hbox{-} {\widehat{\mathrm{S}}}_{\mathrm{X}\le \mathrm{c}}\left(\uptau \right)\right)\cdot {\mathrm{n}}_{\mathrm{X}\le c}+\left(1\hbox{-} {\widehat{\mathrm{S}}}_{\mathrm{X}>c}\left(\uptau \right)\right)\cdot {\mathrm{n}}_{\mathrm{X}>c}} $$

and

9 $$ \widehat{\mathrm{S}\mathrm{P}}\left(\mathrm{c}\right)=\frac{{\widehat{\mathrm{S}}}_{\mathrm{X}\le \mathrm{c}}\left(\uptau \right)\cdot {\mathrm{n}}_{\mathrm{X}\le c}}{{\widehat{\mathrm{S}}}_{\mathrm{X}\le \mathrm{c}}\left(\uptau \right)\cdot {\mathrm{n}}_{\mathrm{X}\le c}+{\widehat{\mathrm{S}}}_{\mathrm{X}>c}\left(\uptau \right)\cdot {\mathrm{n}}_{\mathrm{X}>c}}\ . $$

As an alternative, one could use the indirect estimators of SE(c) and SP(c) based on nearest neighbor estimates of the joint survival between X and Z [12]. This could have the advantage to relax the assumption of independence completely at random between C and Z since it requires only conditional independence given X.

The investigated ROC-based cut-point finding methods could be now easily extended to the censored failure time outcome scenario by plugging-in sample estimates (8) and (9) into objective functions (3), (4) and (5). The optimal cut-point estimates ĉ_J_, ĉ_CZ_ and ĉ_ER_ are then obtained by maximizing the objective functions (3), (4) and (5) over all possible cut-point values c of X [8].

It is worth of note that although the three methods point theoretically to the same cut-point under Normal homoscedastic distributions of the biomarker in diseased and disease-free subjects, the corresponding sample estimators here presented do not lead necessarily to the same estimated cut-point in a single sample. This motivates the estimator performance comparison presented in the next section.

### Simulation protocol

We conducted a simulation study to compare the performance of the Youden index (3), the concordance probability (4) and the point closest-to-(0,1) corner in the ROC plane (5) methods in the estimation of the optimal cut-point in a censored failure time outcome scenario.

Data were simulated as follows:

- The time-to-event Z was generated according to an exponential survival function S(t) = 1 − e^− 2t^.
- τ was set equal to 0.35 in order to achieve a disease fraction of 50% and to 0.20, 0.14, 0.11 and 0.08 to achieve disease fractions of 33%, 25%, 20% and 15%.
- Depending on whether Z ≤ τ or Z > τ, the biomarker X was generated according to N(μ_Z≤τ_, 1) or N(0,1), respectively. This leads to SE(c) = 1 − Φ(c − μ_Z ≤ τ_) and SP(c) = Φ (c), where Φ denotes the standard Normal distribution function. It has been previously shown that within this scenario the objective functions (3), (4) and (5) reach their maximum in correspondence of the same true cut-point, i.e. c_opt_ = μ_Z ≤ τ_/2 [2,5]. Analytically, this common cut-point occurs at the intersection between the Normal probability density functions of diseased, i.e. f_Z>τ_ (c), and disease-free subjects, i.e. f_Z>*τ*_ (c).
- Similarly, X was generated according to G(2.5, β_Z≤τ_) and G(1.5,1) depending on whether Z ≤ τ or Z > τ. This implies that $\mathrm{S}\mathrm{E}\left(\mathrm{c}\right)=\frac{1}{\Gamma (2.5){\left({\upbeta}_{\mathrm{Z}\le \uptau}\right)}^{2.5}}{\displaystyle {\int}_{\mathrm{c}}^{\infty }{\mathrm{x}}^{1.5}{\mathrm{e}}^{\hbox{-} \mathrm{x}/{\upbeta}_{\mathrm{Z}\le \uptau}}\mathrm{d}\mathrm{x}}$ and $\mathrm{S}\mathrm{P}\left(\mathrm{c}\right)\kern0.5em =\kern0.5em \frac{1}{\Gamma (1.5)}{\displaystyle {\int}_{-\infty}^{\mathrm{c}}{\mathrm{x}}^{1.5}{\mathrm{e}}^{-\mathrm{x}}\mathrm{d}\mathrm{x}}$. Within this scenario, the objective functions (3), (4) and (5) point to different true cut-points, and a closed form for c_opt_ cannot be derived [2,5].
- μ_Z≤τ_ was set equal to {0.51, 1.05, 1.68, 2.56} and β_Z≤τ_ to {0.79, 1.22, 1.97, 3.82} in order to achieve a wide variety of the classification accuracy, i.e. J(c_J_) = {0.2, 0.4, 0.6, 0.8}, CZ(c_CZ_) = {0.36, 0.49, 0.64, 0.81}, ER(c_ER_) = {0.57, 0.42, 0.28, 0.14}, ranging from a poor one (J = 0.2, CZ = 0.36 and ER = 0.57) to a high one (J = 0.8, CZ = 0.81 and ER = 0.14).
- The simulation of the survival times first, and thereafter the biomarker values, is somehow counterintuitive since the “natural” ordering suggests that time should be generated depending on the biomarker values, and not vice versa. This choice was done only to keep directly under control the theoretical values of SE and SP, and thus of the three objective functions, Youden index (3), concordance probability (4) and point closest-to-(0,1) corner (5).
- To simulate independent censoring, the censoring time C was generated according to a uniform distribution in the interval [0,b]. When we considered the scenario with a disease fraction equal to 50%, b was set equal to 2, 1 and 0.66 time units in order to achieve different censoring levels, i.e. 12%, 25% and 38%. Within the scenarios with disease fraction equal to 33%, 25%, 20% and 15%, b was set equal to 0.67, 0.50, 0.40 and 0.29 time units to achieve a censoring level of 25%.
- The observed survival data was calculated by T = min(Z,C) and δ = 1 if T = Z and δ = 0 if T = C.

We generated 1000 samples {(X_i_, T_i_, δ_i_); i = 1, …, N} of size N = 50, N = 100, N = 200 and N = 400 with a disease fraction of 50% and three different censoring levels, i.e. 12%, 25% and 38%. Moreover, we generated 1000 samples of size N = 100, N = 150, N = 200 and N = 250 with different disease fractions, i.e. 33%, 25%, 20% and 15%, and a censoring level of 25%. For each sample, we determined by empirical numerical maximization [2,5,8] the optimal cut-point estimates ĉ_J_, ĉ_CZ_ and ĉ_ER_ for the Youden index, the concordance probability and the point closest-to-(0,1) corner in the ROC plane methods, respectively. The relative bias and the mean square error (MSE) of each method were computed by E[(ĉ_._ − c)] and E[(ĉ_._ − c)^2^], where the expectation was meant to be the average over the N simulated samples.

Given the computational burden, we applied the bootstrap resampling technique to estimate the standard deviation and the confidence interval (CI) for the optimal cut-point for some selected scenarios with sample size N = 100 or N = 150. We applied the Efron and Tibshirani’s procedure [13] as follow:

- From each sample {(X_i_, T_i_, δ_i_); i = 1, …, N}, we applied a random sampling with replacement to draw 200 bootstrap samples in order to calculate the bootstrap estimate ĉ_B_ (B = 1, …, 200).
- We applied the basic percentile method, taking the 0.025 and 0.975 percentiles of the ĉ_B_ bootstrap distribution in order to construct a 95% CI of the optimal cut-point within each of the 1000 generated samples. Each bootstrap sample contributed one cut-point estimate, so that the standard deviation of the 200 cut-point estimates was used as the bootstrap estimator of the standard deviation (SD_B_) for the estimated cut-point.
- The CI for the cut-point for each of the investigated methods was subsequently evaluated by computing coverage probability and mean length.

Simulations have been performed in R version 2.15 [14].

## Results

### Simulation study

The results of the simulation exercises under Normal homoscedastic distribution of X with a diseased and disease-free fraction of 50% are shown in Tables 1, 2 and 3 for different censoring levels, i.e. 12%, 25% and 38%, respectively. The relative bias of the investigated methods is small on all levels of classification accuracy, except for the scenario with J = 0.2 and CZ = 0.36 for samples of size N = 50 and N = 100, and it increases as the censoring level increases. By comparing the MSEs, it can be noticed that the point closest-to-(0,1) corner in the ROC plane and the concordance probability methods have better performance than the Youden index method. Indeed, the MSE is inversely related to sample size and it increases as the censoring level increases. The performance of the investigated methods improves with increasing classification accuracy.

**Table 1:** **Relative bias and Mean Square Error (MSE) of the cut-point in the normal homoscedastic scenario** ^**†**^ **with diseased and disease-free fractions of 50% and a censoring level of 12%**

				**Youden index**		**Concordance probability**		**Point closest-to-(0,1) corner**	
**J(c**_**opt**_ **)** ^**‡**^	**CZ(c**_**opt**_ **)** ^**‡**^	**c**_**opt**_	**N**	**Relative bias**	**MSE**	**Relative bias**	**MSE**	**Relative bias**	**MSE**
**0.2**	**0.36**	0.25	50	0.1613	0.3046	0.1503	0.1264	0.1854	0.0891
			100	0.1168	0.2226	0.0687	0.0719	0.0769	0.0513
			200	0.0696	0.1750	0.0546	0.0501	0.0589	0.0360
			400	0.0813	0.1137	0.0507	0.0273	0.0404	0.0199
**0.4**	**0.49**	0.52	50	0.0793	0.1870	0.0623	0.1158	0.0618	0.0785
			100	0.0584	0.1295	0.0346	0.0724	0.0356	0.0462
			200	0.0038	0.0870	0.0009	0.0462	0.0032	0.0279
			400	0.0073	0.0536	0.0070	0.0270	0.0100	0.0147
**0.6**	**0.64**	0.84	50	0.0724	0.1378	0.0599	0.1157	0.0555	0.0763
			100	0.0311	0.0884	0.0350	0.0675	0.0259	0.0421
			200	0.0147	0.0575	0.0201	0.0408	0.0152	0.0227
			400	0.0086	0.0332	0.0015	0.0240	−0.0009	0.0138
**0.8**	**0.81**	1.28	50	0.0568	0.1203	0.0531	0.1117	0.0528	0.0880
			100	0.0399	0.0727	0.0379	0.0669	0.0291	0.0465
			200	0.0167	0.0435	0.0149	0.0383	0.0115	0.0241
			400	0.0033	0.0290	0.0021	0.0253	0.0027	0.0148

^†^X_Z ≤ τ_ ~ N(μ_Z ≤ τ_, 1), X_Z >τ_ ~ N(0, 1). ^‡^The levels of J and CZ are achieved by μ_Z ≤ τ_ = 0.51, 1.05, 1.68, 2.56, respectively.

**Table 2:** **Relative bias and Mean Square Error (MSE) of the cut-point in the normal homoscedastic scenario** ^**†**^ **with diseased and disease-free fractions of 50% and a censoring level of 25%**

				**Youden index**		**Concordance probability**		**Point closest-to-(0,1) corner**	
**J(c**_**opt**_ **)** ^**‡**^	**CZ(c**_**opt**_ **)** ^**‡**^	**c**_**opt**_	**N**	**Relative bias**	**MSE**	**Relative bias**	**MSE**	**Relative bias**	**MSE**
**0.2**	**0.36**	0.25	50	0.1327	0.3204	0.1582	0.1366	0.1498	0.0983
			100	0.1172	0.2448	0.1078	0.0798	0.1016	0.0565
			200	0.0777	0.1951	0.0796	0.0525	0.0561	0.0388
			400	0.1123	0.1196	0.0541	0.0304	0.0408	0.0214
**0.4**	**0.49**	0.52	50	0.0728	0.2029	0.0740	0.1202	0.0739	0.0793
			100	0.0490	0.1332	0.0327	0.0771	0.0339	0.0489
			200	0.0200	0.0946	0.0101	0.0522	0.0031	0.0294
			400	0.0042	0.0573	0.0037	0.0274	0.0075	0.0157
**0.6**	**0.64**	0.84	50	0.0651	0.1446	0.0589	0.1182	0.0477	0.0817
			100	0.0491	0.0947	0.0428	0.0428	0.0355	0.0355
			200	0.0185	0.0605	0.0237	0.0447	0.0192	0.0237
			400	0.0058	0.0367	0.0055	0.0255	0.0004	0.0148
**0.8**	**0.81**	1.28	50	0.0514	0.1236	0.0523	0.1158	0.0473	0.0990
			100	0.0384	0.0786	0.0373	0.0722	0.0299	0.0505
			200	0.0149	0.0464	0.0140	0.0401	0.0081	0.0259
			400	0.0053	0.0314	0.0064	0.0271	0.0024	0.0158

^†^X_Z ≤ τ_ ~ N(μ_Z ≤ τ_, 1), X_Z >τ_ ~ N(0, 1). ^‡^The levels of J and CZ are achieved by μ_Z ≤ τ_ = 0.51, 1.05, 1.68, 2.56, respectively.

**Table 3:** **Relative bias and Mean Square Error (MSE) of the cut-point in the normal homoscedastic scenario** ^**†**^ **with diseased and disease-free fractions of 50% and a censoring level of 38%**

				**Youden index**		**Concordance probability**		**Point closest-to-(0,1) corner**	
**J(c**_**opt**_ **)** ^**‡**^	**CZ(c**_**opt**_ **)** ^**‡**^	**c**_**opt**_	**N**	**Relative bias**	**MSE**	**Relative bias**	**MSE**	**Relative bias**	**MSE**
**0.2**	**0.36**	0.25	50	0.1345	0.3419	0.1918	0.1514	0.1812	0.1099
			100	0.1347	0.2592	0.1042	0.0894	0.1225	0.0627
			200	0.0547	0.2084	0.0638	0.0572	0.0652	0.0413
			400	0.0932	0.1395	0.0177	0.0350	0.0200	0.0245
**0.4**	**0.49**	0.52	50	0.0912	0.2147	0.0829	0.1303	0.0624	0.0891
			100	0.0595	0.1418	0.0460	0.0841	0.0438	0.0518
			200	0.0106	0.1039	0.0082	0.0541	0.0024	0.0339
			400	0.0085	0.0641	−0.0021	0.0314	0.0009	0.0173
**0.6**	**0.64**	0.84	50	0.0446	0.1596	0.0560	0.1262	0.0507	0.0890
			100	0.0628	0.1094	0.0599	0.0794	0.0492	0.0494
			200	0.0258	0.0653	0.0256	0.0492	0.0212	0.0260
			400	0.0042	0.0408	0.0052	0.0287	0.0012	0.0160
**0.8**	**0.81**	1.28	50	0.0461	0.1404	0.0435	0.1340	0.0310	0.1186
			100	0.0449	0.0883	0.0411	0.0792	0.0282	0.0587
			200	0.0132	0.0503	0.0108	0.0462	0.0073	0.0297
			400	0.0039	0.0367	0.0041	0.0323	0.0030	0.0185

^†^X_Z ≤ τ_ ~ N(μ_Z ≤ τ_, 1), X_Z >τ_ ~ N(0, 1). ^‡^The levels of J and CZ are achieved by μ_Z ≤ τ_ = 0.51, 1.05, 1.68, 2.56, respectively.

Table 4 shows the results under Normal homoscedastic distribution of X when considering different disease fractions and a censoring level of 25%. The relative bias of the investigated methods is small on all levels of classification accuracy, except for the scenarios with a disease fraction equal to 15%. As above, the point closest-to-(0,1) corner in the ROC plane and the concordance probability methods outperform the Youden index method. The MSE is lower for the point closest-to-(0,1) corner in the ROC plane method, too.

**Table 4:** **Relative bias and Mean Square Error (MSE) of the cut-point in the normal homoscedastic scenario** ^**†**^ **with different disease fractions and a censoring level of 25%**

					**Youden index**		**Concordance probability**		**Point closest-to-(0,1) corner**	
**J(c**_**opt**_)^**‡**^	**CZ(c**_opt_ **)** ^‡^	**c**_opt_	_N_	**Disease fraction**	**Relative bias**	**MSE**	**Relative bias**	**MSE**	**Relative bias**	**MSE**
**0.2**	**0.36**	0.25	100	15%	0.2296	0.3298	0.4150	0.1390	0.4114	0.1060
			150	33%	0.2188	0.2089	0.1921	0.0611	0.1749	0.0440
			200	25%	0.0973	0.2125	0.1758	0.0666	0.1822	0.0482
			250	20%	0.1872	0.1871	0.2246	0.0592	0.2164	0.0421
**0.4**	**0.49**	0.52	100	15%	0.1389	0.1944	0.2084	0.1320	0.2450	0.1051
			150	33%	0.0786	0.1048	0.0625	0.0575	0.0741	0.0370
			200	25%	0.0516	0.1147	0.0701	0.0606	0.0674	0.0381
			250	20%	0.0926	0.0990	0.0951	0.0528	0.0940	0.0346
**0.6**	**0.64**	0.84	100	15%	0.1457	0.1658	0.1503	0.1366	0.1765	0.1101
			150	33%	0.0522	0.0778	0.0547	0.0590	0.0453	0.0367
			200	25%	0.0500	0.0744	0.0516	0.0512	0.0515	0.0286
			250	20%	0.0543	0.0742	0.0585	0.0569	0.0596	0.0344
**0.8**	**0.81**	1.28	100	15%	0.1219	0.1534	0.1283	0.1478	0.1337	0.1274
			150	33%	0.0401	0.0702	0.0439	0.0645	0.0387	0.0432
			200	25%	0.0360	0.0568	0.0375	0.0513	0.0438	0.0364
			250	20%	0.0535	0.0641	0.0558	0.0585	0.0535	0.0390

^†^X_Z ≤ τ_ ~ N(μ_Z ≤ τ_, 1), X_Z >τ_ ~ N(0, 1). ^‡^The levels of J and CZ are achieved by μ_Z ≤ τ_ = 0.51, 1.05, 1.68, 2.56, respectively.

The results of the simulation exercise under Gamma distribution of X when considering a diseased and disease-free fraction of 50% and a censoring level of 25% are shown in Table 5. In such scenario, the three objective functions point to different cut-points, and only a relative performance comparison could be made. We note that methods’ performance improve with increasing classification accuracy in terms of relative bias, and also that the Youden index method showed an unsatisfactory performance in the scenario with J = 0.2 and CZ = 0.36 for samples of size N = 50 and N = 100. The MSE is inversely related to sample size but it increases as the classification accuracy increases. As in the Normal scenarios, the MSE is lower for the point closest-to-(0,1) corner in the ROC plane method.

**Table 5:** **Relative Bias and Mean Square Error (MSE) of the cut-point in the Gamma scenario** ^**†**^ **with diseased and disease-free fraction of 50% and a censoring level of 25%**

						**Youden index**		**Concordance probability**		**Point closest-to-(0,1) corner**	
**J(c**_**opt**_ **)** ^**‡**^	**CZ(c**_**opt**_ **)** ^**‡**^	**c**_**J**_	**c**_**CZ**_	**c**_**ER**_	**N**	**Relative bias**	**MSE**	**Relative bias**	**MSE**	**Relative bias**	**MSE**
**0.2**	**0.36**	1.12	1.35	1.38	50	0.2038	0.3822	0.0532	0.1584	0.0464	0.1235
					100	0.1605	0.2987	0.0429	0.0975	0.0352	0.0712
					200	0.0840	0.1825	0.0138	0.0638	0.0087	0.0446
					400	0.0668	0.1156	0.0105	0.0348	0.0086	0.0261
**0.4**	**0.49**	1.79	1.81	1.82	50	0.0863	0.4446	0.0605	0.2700	0.0473	0.1771
					100	0.0460	0.2585	0.0408	0.1529	0.0311	0.1001
					200	0.0267	0.1740	0.0136	0.0924	0.0059	0.0521
					400	0.0144	0.1183	0.0133	0.0571	0.0062	0.0346
**0.6**	**0.64**	2.45	2.41	2.36	50	0.0391	0.5201	0.0411	0.4108	0.0383	0.2741
					100	0.0297	0.3288	0.0293	0.2380	0.0267	0.1536
					200	0.0186	0.2117	0.0170	0.1539	0.0138	0.0815
					400	0.0067	0.1433	0.0077	0.0941	0.0081	0.0445
**0.8**	**0.81**	3.42	3.38	3.24	50	0.0610	0.9808	0.0663	0.9103	0.0782	0.7477
					100	0.0526	0.5678	0.0508	0.5156	0.0429	0.3593
					200	0.0210	0.3317	0.0241	0.3039	0.0166	0.1739
					400	0.0090	0.1929	0.0107	0.1693	0.0079	0.0885

^†^X_Z ≤ τ_ ~ G(2.5, β_Z ≤ τ_), X_Z >τ_ ~ G(1.5, 1). ^‡^The levels of J and CZ are achieved by β_Z ≤ τ_ = 0.79, 1.22, 1.97, 3.82, respectively.

Bootstrap standard deviation, coverage probability and mean length of the 95% bootstrap CI for the cut-point are shown in Additional file 1: Table S1 for some selected simulation scenarios under Normal homoscedastic distribution of X. The SD_B_ of the point closest-to-(0,1) corner in the ROC plane approach is lower than the SD_B_ of the Youden index and concordance probability methods. Coverage probabilities are fluctuating around the nominal level. 95% bootstrap CIs were narrower when considering the scenarios with better classification accuracies, i.e. J of 0.6 and 0.8.

### Applicative example on acute lymphoblastic leukemia

**Figure 2:**
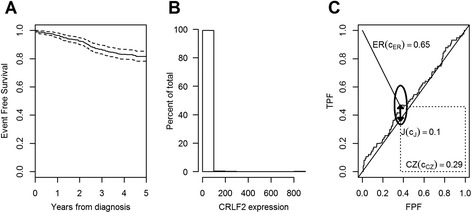
Applicative example on acute lymphoblastic leukemia. Panel **A**. Estimated survival curve with 95% confidence bands. Panel **B**. Histogram for the CRLF2 expression. Panel **C**. ROC curve for the CRLF2 biomarker with the three objective functions: the Youden index J(ĉ_J_) represented by the thick line segment, the concordance probability CZ(ĉ_CZ_) represented by the area of the dotted rectangle and the distance from the (0,1) corner represented by the thin line segment. The 95% elliptic asymptotic confidence interval of (FPF(ĉ), TPF(ĉ)) is also represented.

Acute lymphoblastic leukemia (ALL) is the most common malignancy in children and it presents, in the large majority (70%), a B-cell precursor (BCP) ALL immunophenotype. The cure rate of BCP-ALL is nowadays higher than 80%, but the probability of survival of patients who relapse is only 40% [10]. Recent studies had reported that a higher expression of the *cytokine receptor-like factor 2* (CRLF2) was associated to a higher risk of relapse. In their study, Palmi et al. [10] aimed at defining a cut-point for the CRLF2, as measured at diagnosis, that would allow to identify those children more likely to relapse in order to be able to tailor upfront the treatment intensity in future protocols. We applied the presented methods to this study that includes 464 Italian BCP-ALL children enrolled (from February 2003 to July 2005) in the AIEOP (*Associazione Italiana Ematologia Oncologia Pediatrica*) treatment protocol “AIEOP-BFM ALL2000”. The time window of interest for predicting relapse was of 5 years, and in that time frame 74 relapses had been observed over a total of 79 relapses in the cohort. Figure 2 Panel A shows the event free survival (EFS) curve along with the 95% confidence bands. The 5-year EFS estimate was 81.6% (95% CI, 78.1%-85.1%). The CRLF2 expression had a right-skewed distribution (Shapiro-Wilk normality test P < 0.01) ranging from 0.006 to 810-fold change compared to the overall median value (Figure 2 Panel B) [10].

The estimated cut-point for the CRLF2 expression was ĉ = 1.46, the same for all three methods, but the classification accuracy of this biomarker was very low, as depicted by the ROC curve and expressed by the Youden index J = 0.10 calculated for the identified cut-point (Figure 2 Panel C). The 95% bootstrap (999 replicates) CI estimates for the cut-point are (0.12, 21.61), (0.70, 1.98) and (0.70, 1.86), for the Youden index, the concordance probability and the point closest-to-(0,1) corner in the ROC plane methods, respectively. The 95% delta-method based elliptic asymptotic confidence interval of (FPF(ĉ), TPF(ĉ)) is represented in Figure 2 Panel C. This interval is elliptic in the logit space since it was obtained from a joint interval on the logit transformation of $\widehat{\mathrm{TPF}}$ and $\widehat{\mathrm{FPF}},$ which are correlated, altough modestly, since censored observations contribute to both estimators [9].

### Applicative example on primary biliary cirrhosis

**Figure 3:**
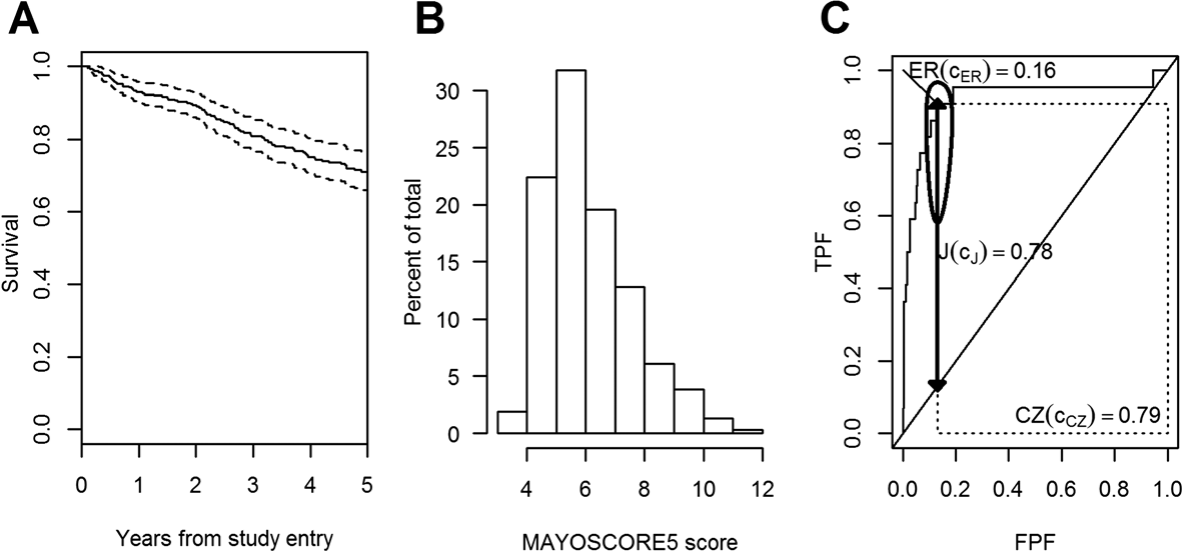
Applicative example on primary biliary cirrhosis. Panel **A**. Estimated survival curve with 95% confidence bands. Panel **B**. Histogram for the MAYOSCORE5 prognostic score. Panel **C**. ROC curve for the MAYOSCORE5 prognostic score with the three objective functions: the Youden index J(ĉ_J_) represented by the thick line segment, the concordance probability CZ(ĉ_CZ_) represented by the area of the dotted rectangle and the distance from the (0,1) corner ER(ĉ_ER_) represented by the thin line segment. The 95% elliptic asymptotic confidence interval of (FPF(ĉ), TPF(ĉ)) is also represented.

We used data, made available online inside the survivalROC R package [15], from a randomized placebo-controlled trial of D-penicilliamine (DPCA) for the treatment of primary biliary cirrhosis (PBC), conducted at the Mayo Clinic between 1974 and 1984. Among the 312 subjects randomized to the study, 125 died by the end of the follow-up. The survival curve is shown in Figure 3 Panel A. Data from this negative study were used to develop a clinical prediction model for mortality based on bilirubin and albumin levels, prothrombin time, presence of edema and age at diagnosis [11]. We aimed to find a cut-point for this widely used prognostic score (hereafter named MAYOSCORE5) by considering a time frame of one year, i.e. τ = 365 days, from study entry, by when 22 deaths occurred. The MAYOSCORE5 score ranged between 3.74 and 11.250 with a median of 5.75, and its distribution is quite symmetric (Figure 3 Panel B), even if not Normal according to a formal test (Shapiro-Wilk normality test P < 0.01). The three investigated methods, i.e. the Youden index, the concordance probability and the point closest-to-(0,1) corner in the ROC plane, lead to the same estimated cut-point ĉ = 7.35. The biomarker had a good classification accuracy, with a Youden index J = 0.78 and a concordance probability CZ = 0.79, as shown in Figure 3. The bootstrap (999 replicates) standard deviations for ĉ are 0.11, 0.09 and 0.05 for the Youden index, concordance probability and point closest-to-(0,1) corner in the ROC plane methods, respectively. Moreover, the 95% bootstrap CI estimates for the cut-point are (6.99, 7.35), (7.03, 7.35) and (7.30, 7.48), for the Youden index, the concordance probability and the point closest-to-(0,1) corner in the ROC plane methods, respectively. The 95% delta-method based elliptic asymptotic confidence interval of (FPF(ĉ), TPF(ĉ)) is represented in Figure 3 Panel C. This interval is elliptic in the logit space since it was obtained from a joint interval on the logit transformation of $\widehat{\mathrm{TPF}}$ and $\widehat{\mathrm{FPF}},$ which are correlated, altough modestly, since censored observations contribute to both estimators [9].

## Discussion

In this work we extended three widely used ROC-based methods for defining a cut-point of a continuous biomarker, namely the Youden index [1], the concordance probability [2,3] and the point closest to-(0,1) corner [4], to the censored failure time outcome by using non-parametric estimators of sensitivity and specificity in the presence of censoring [9]. The minimum p-value approach [6] was not extended to the censored data setting since its objective function is computed under the null hypothesis of absence of association between the true binary status and the biomarker classification, in contrast with the presence of some discrimination potential that leads to the dichotomization issue itself. In fact, this last method showed an unsatisfactory performance when oriented to identify a cut-point in the presence of a binary outcome [5]. The same consideration would also apply to other test-based methods such as the log-rank, which in addition it is not specifically related to a predefined time horizon [16].

The simulation protocol was set in order to keep directly under control the theoretical values of sensitivity and specificity by simulating the survival times first, and afterwards the biomarker values conditional on time. This strategy can be however reversed by working with the Bayes’ theorem.

We mainly considered the case where the three methods identify theoretically the same underlying true cut-point, as in the presence of Gaussian homoscedastic biomarker distributions. The main issue a researcher faces in this common situation is the choice between alternative estimators of the same parameter (cut-point). We showed that the point closest-to-(0,1) corner approach has the best performance from simulations in terms of mean square error and relative bias. However, the calculation of the Youden index [1] or the concordance probability [2,3] associated to the cut-point identified through the point closest-to-(0,1) corner estimator could be used to ease interpretability and to communicate the classification performance of the biomarker given the lack of clinical meaning of the point closest-to-(0,1) corner objective function [4].

In the absence of a closed form, we provided estimation of the standard deviation and 95% confidence interval for the cut-point by the bootstrap method [13]. We used only 200 replicates due to computational burden of the simulation exercise. This may have led to coverage under the nominal level in some scenarios. We recommend to use a larger number of replicates in real data applications. In the applications presented in this paper, we used 999 bootstrap replicates. In addition, the achieved performance of the dichotomized biomarker classification associated to the estimated cut-point can be represented through a confidence interval of the point on the ROC curve [9,17].

It should also pointed out that a good estimation of the cut-point did not necessary lead to a good estimation of the corresponding objective functions, and vice versa [8]. In our simulation scenario, we found an overestimation of the Youden index and concordance probability, and an underestimation of the closest-to-(0,1) corner objective function, at the optimal estimated cut-point. The bias decreased with increasing sample size and classification accuracy of the biomarker. This is due to the fact that most properties of estimators, such as bias, are not preserved under non-linear monotonic transformations [8]. Thus, when communicating the clinical value of an identified cut-point, we also recommend to provide the confidence interval estimate of the associated objective function. For the Youden index method, the variability of the objective function estimate in the presence of censored data can be addressed by applying the delta method and by handling the covariance issue as in Antolini and Valsecchi [9].

When methods point to different true cut-points, as in the Gamma distribution scenario, since the parameters of interest are different, estimators cannot be solely chosen relying on performance. In this case, scientists should rather choose according to the meaning of the underlying objective functions. For instance, the Youden index method [1] could be chosen if the researcher is interested in interpreting the net gain of the true positive fraction accounting for the false positive fraction, while the concordance probability approach [2,3] could be used if the researcher aims to interpret the probability of being below or above the cut-point for any random pair of disease-free and diseased subjects. When the focus is not on a specific time horizon, other cut-point finding methods could be considered, such as Harrell’s C, or even model-based derived indicators [16].

When disease prevalence is far from 50%, as in many applications, the three investigated methods could be modified by a weighting system in order to take into account the relative importance attributed to a true positive or a true negative result by addressing aspects related to both disease prevalence and patient’s benefit associated with a correct positive test result [18,19]. Moreover, in a setting with a high or poor overall survival, the estimated cut-point may have larger variation [3]. It has also to be considered that besides the relatively high/poor overall survival, the investigated objective functions do not generally lead to optimal cut-points on the boundary of the biomarker distribution [5]. This is nice, since a cut-point on the boundary could indeed lead to a very limited sample size for the estimation of one of the two conditional survivals which are plugged into sensitivity and specificity.

The proposed example on CRLF2 expression in acute lymphoblastic leukemia [10] shows that in some clinical applications methods based on sensitivity and specificity may lead to unsatisfactory cut-points due to a moderate discrimination potential of the biomarker, as represented by the whole ROC curve. By contrast, the application example on the Mayo score predicting mortality in primary biliary cirrhosis shows a satisfactory result.

Future works should address the issue of when cut-point finding should be based on predictive values [20], more appealing for clinical interpretation and use, rather than on sensitivity and specificity. If the point of a biomarker based test is to use it to discriminate prognosis, clinicians need to know the probability that the outcome will be favourable or unfavourable given the test outcome. In this way, clinicians would approach the data from the direction of the test results, using predictive values [21], although their cut-point definition would be influenced by the prevalence of the condition. For example, when predictive values had been used to detect a cut-point for the CRLF2, as done in the original paper [10], a very extreme value of the cut-point would have been identified, which defines a very rare subgroup with a high risk of relapse.

## Conclusions

We showed the extension of the Youden index, the concordance probability and the point closest to-(0,1) corner in the ROC plane cut-point finding methods to the case of censored failure time outcome. When considering the Normal homoscedastic scenario where the investigated methods lead to the same cut-point, the point closest-to-(0,1) corner approach has the best performance from simulations in terms of mean square error and relative bias. However, we discuss the use of the Youden index or the concordance probability associated to the cut-point identified through the closest-to-(0,1) corner approach to ease interpretability of the classification performance of the biomarker.

## Additional file

Additional file 1: Table S1. Bootstrap standard deviation, coverage probability and mean length of the 95% confidence interval of the cut-point in the normal homoscedastic scenario with different disease fractions and censoring levels.
