# Supplementary material for: Optimal cut-point definition in biomarkers: the case of censored failure time outcome
Source: BMC Med Res Methodol. 2015 Mar 21;15:24. doi: 10.1186/s12874-015-0009-y (PMC4430986; doi:10.1186/s12874-015-0009-y)
Supplement: Additional file 1: Table S1. — Bootstrap standard deviation, coverage probability and mean length of the 95% confidence interval of the cut-point in the normal homoscedastic scenario with different disease fractions and censoring levels. [file 12874_2015_9_MOESM1_ESM.docx]

**Additional file 1: Table S1** Bootstrap standard deviation, coverage probability and mean length of the 95% confidence interval estimation of the Youden index, concordance probability and point closest-to-(0,1)-corner in the ROC plane estimators. The normal homoscedastic scenario, with different disease fractions and censoring levels^†^.

|  | | | | | **Youden Index** | | | **Concordance probability** | | | **Point closest-to-(0,1) corner** | | |
| --- | --- | --- | --- | --- | --- | --- | --- | --- | --- | --- | --- | --- | --- |
| **J(c_opt_)** ^‡^ | **CZ(c_opt_)** ^‡^ | **N** | **Disease fraction** | **Censoring level** | **SD_B_** | **Coverage** | **Mean Length** | **SD_B_** | **Coverage** | **Mean Length** | **SD_B_** | **Coverage** | **Mean Length** |
| **0.2** | **0.36** | 100 | 50% | 12% | 0.4606 | 0.968 | 1.6188 | 0.2844 | 0.964 | 1.0068 | 0.2409 | 0.960 | 0.8508 |
|  |  | 100 | 50% | 38% | 0.4758 | 0.966 | 1.6586 | 0.3086 | 0.956 | 1.0881 | 0.2589 | 0.954 | 0.9158 |
|  |  | 150 | 33% | 25% | 0.4330 | 0.961 | 1.5257 | 0.2609 | 0.930 | 0.9143 | 0.2188 | 0.923 | 0.7741 |
| **0.4** | **0.49** | 100 | 50% | 12% | 0.3504 | 0.961 | 1.2307 | 0.2677 | 0.956 | 0.9371 | 0.2158 | 0.943 | 0.7626 |
|  |  | 100 | 50% | 38% | 0.3673 | 0.968 | 1.2888 | 0.2860 | 0.948 | 0.9917 | 0.2316 | 0.948 | 0.8178 |
|  |  | 150 | 33% | 25% | 0.3235 | 0.954 | 1.1433 | 0.2448 | 0.938 | 0.8616 | 0.1961 | 0.927 | 0.6941 |
| **0.6** | **0.64** | 100 | 50% | 12% | 0.2876 | 0.958 | 0.9936 | 0.2552 | 0.954 | 0.8880 | 0.2064 | 0.938 | 0.7228 |
|  |  | 100 | 50% | 38% | 0.3006 | 0.953 | 1.0473 | 0.2689 | 0.943 | 0.9386 | 0.2212 | 0.939 | 0.7712 |
|  |  | 150 | 33% | 25% | 0.2699 | 0.939 | 0.9309 | 0.2371 | 0.928 | 0.8177 | 0.1876 | 0.917 | 0.6569 |
| **0.8** | **0.81** | 100 | 50% | 12% | 0.2565 | 0.920 | 0.8753 | 0.2469 | 0.918 | 0.8405 | 0.2110 | 0.905 | 0.7287 |
|  |  | 100 | 50% | 38% | 0.2725 | 0.917 | 0.9324 | 0.2641 | 0.916 | 0.9024 | 0.2375 | 0.908 | 0.8307 |
|  |  | 150 | 33% | 25% | 0.2461 | 0.878 | 0.8350 | 0.2362 | 0.880 | 0.8030 | 0.1967 | 0.864 | 0.6756 |

^†^ $X_{Z\leq\tau}\sim N(\mu_{Z\leq\tau},1)$, $X_{Z>\tau}\sim N(0,1)$. ^‡^The levels of J and CZ are achieved by $\mu_{Z\leq\tau}$= 0.51, 1.05, 1.68, 2.56, respectively.
